# Folate intake, alcohol consumption, and the methylenetetrahydrofolate reductase (*MTHFR*) C677T gene polymorphism: influence on prostate cancer risk and interactions

**DOI:** 10.3389/fonc.2012.00100

**Published:** 2012-08-14

**Authors:** Lindsay C. Kobayashi, Heather Limburg, Qun Miao, Christy Woolcott, Leanne L. Bedard, Thomas E. Massey, Kristan J. Aronson

**Affiliations:** ^1^Department of Community Health and Epidemiology, Queen's UniversityKingston, ON, Canada; ^2^Division of Cancer Care and Epidemiology, Queen's Cancer Research Institute, Queen's UniversityKingston, ON, Canada; ^3^Perinatal Epidemiology Research Unit, Departments of Obstetrics and Gynecology and Pediatrics, Dalhousie UniversityHalifax, NS, Canada; ^4^Pharmacology and Toxicology Graduate Program, Department of Biomedical and Molecular Sciences, Queen's UniversityKingston, ON, Canada

**Keywords:** prostate cancer, folate, alcohol, genetic variants, carbon metabolism, gene-environment interactions, case-control study, male health

## Abstract

**Purpose:** Folate is essential to DNA methylation and synthesis and may have a complex dualistic role in prostate cancer. Alcohol use may increase risk and epigenetic factors may interact with lifestyle exposures. We aimed to characterize the independent and joint effects of folate intake, alcohol consumption, and the *MTHFR* C677T gene polymorphism on prostate cancer risk, while accounting for intakes of vitamins B_2_, B_6_, B_12_, methionine, total energy, and confounders. **Methods:** A case-control study was conducted at Kingston General Hospital of 80 incident primary prostate cancer cases and 334 urology clinic controls, all with normal age-specific PSA levels (to exclude latent prostate cancers). Participants completed a questionnaire on folate and alcohol intakes and potential confounders prior to knowledge of diagnosis, eliminating recall bias, and blood was drawn for *MTHFR* genotyping. Joint effects of exposures were assessed using unconditional logistic regression and significance of multiplicative and additive interactions using general linear models. **Results:** Folate, vitamins B_2_, B_6_, B_12_, methionine, and the CT and TT genotypes were not associated with prostate cancer risk. The highest tertile of lifetime alcohol consumption was associated with increased risk (OR = 2.08; 95% CI: 1.12–3.86). Consumption of >5 alcoholic drinks per week was associated with increased prostate cancer risk among men with low folate intake (OR = 2.38; 95% CI: 1.01–5.57), and higher risk among those with the CC *MTHFR* genotype (OR = 4.43; 95% CI: 1.15–17.05). Increased risk was also apparent for average weekly alcohol consumption when accounting for the multiplicative interaction between folate intake and *MTHFR* C677T genotype (OR = 3.22; 95% CI: 1.36–7.59). **Conclusion:** Alcohol consumption is associated with increased prostate cancer risk, and this association is stronger among men with low folate intake, with the CC *MTHFR* genotype, and when accounting for the joint effect of folate intake and *MTHFR* C677T genotype.

## Introduction

Prostate cancer is the second most common malignancy among men (Ferlay et al., 2010). With 5–10% attributable to family history and genetic susceptibility (National Cancer Institute, 2004), etiology of prostate cancer is largely unknown (International Agency for Research on Cancer, 2008). One dietary factor with a complex relationship with prostate cancer is folate, a water-soluble B vitamin that provides substrates for DNA methylation and synthesis of purines and thymidine (Kim, 1999; Fairfield and Fletcher, 2002). Biologic plausibility exists for the contribution of both high and low folate intakes to prostate carcinogenesis. Deficient folate levels have been shown in murine prostate cancer models to result in CpG island hypermethylation and misincorporation of uracil into DNA strands, leading to genetic and epigenetic instability that is characteristic of carcinogenesis (Bistulfi et al., 2010). Conversely, folate deficiency can halt progression of pre-existing tumors in the murine model (Tomaszewski et al., 2011) and high serum folate concentrations have been associated with an increased rate of progression among men with existing prostate cancer (Collin et al., 2010a). Conflicting results from epidemiological research may reflect this potential dualistic role of folate in prostate carcinogenesis (Vlajinac et al., 1997; Weinstein et al., 2003; Hultdin et al., 2005; Pelucchi et al., 2005; Rossi et al., 2006; Stevens et al., 2006; Johansson et al., 2008; Figueiredo et al., 2009; Shannon et al., 2009; Zhang et al., 2009; Beilby et al., 2010; Collin et al., 2010a,b). Understanding of the true effects of folate intake on prostate carcinogenesis is warranted to inform dietary recommendations, especially in the context where many countries have implemented fortification of several foods with folate (Lucock and Yates, 2009).

The effects of folate may be moderated by two potential independent risk factors for prostate cancer: alcohol consumption, and the C677T polymorphism of the methylenetetrahydrofolate reductase (*MTHFR*) gene. A meta-analysis in 2009 showed that consistently heavy alcohol drinking confers higher risk for prostate cancer (Middleton Fillmore et al., 2009). Alcohol directly interferes with the actions of folate in the body by inhibiting its absorption in the small intestine (Dennis, 2000). Heavier drinkers thus have a greater need for folate, which may place them at higher risk for prostate cancer. While some previous epidemiological studies on prostate cancer have controlled for alcohol as a confounding factor, only four have assessed an interaction between folate intake and alcohol consumption, and these have had yielded conflicting results (Weinstein et al., 2003; Pelucchi et al., 2005; Stevens et al., 2006; Shannon et al., 2009).

The *MTHFR* gene encodes the *MTHFR* enzyme that plays an essential role in folate metabolism by catalyzing the production of 5-methyltetrahydrofolate, the main circulating form of folate required for DNA methylation (Lathrop Stern et al., 2000). The C677T (cytosine-to-thymine) mutation of the *MTHFR* gene, CC in wildtype, can produce the heterozygous CT gene variant or the homozygous TT variant: the CT variant occurs in 50% of the population and results in 65% of the wildtype enzyme's functional activity, while the TT variant occurs in approximately 10% of the population and results in 30% functional enzymatic activity (Frosst et al., 1995; Lathrop Stern et al., 2000). The *MTHFR* C677T polymorphism has not been identified in recent genome-wide association studies as associated with susceptibility for prostate cancer (Thomas et al., 2008; Collin et al., 2009; Kote-Jarai et al., 2011; Schumacher et al., 2011). However, it may be relevant when examined in conjunction with folate and alcohol intakes to help explain prostate cancer risk not attributable to susceptibility genes alone. Epidemiological studies have reported associations between the TT variant genotype and reduced risks of colorectal (Tiaoli et al., 2009) and breast (Qi et al., 2010) cancers, while findings regarding prostate cancer conflict (Bai et al., 2009; Collin et al., 2009; Cai et al., 2010). A simulation study supports a role for folate-*MTHFR* genotype interaction in carcinogenesis, showing reduced DNA methylation rates when low *MTHFR* gene activity is combined with high intracellular folate concentrations (Neuhouser et al., 2011). One epidemiological study to date has considered this interaction in prostate cancer, indicating an increased risk associated with high folate intake among men with the TT *MTHFR* genotype (Van Guelpen et al., 2006). To our knowledge, no previous study has examined the interaction between alcohol and *MTHFR* genotype in prostate cancer etiology. We sought to assess the potential independent and joint associations of folate intake, alcohol consumption, and the C677T *MTHFR* gene polymorphism with prostate cancer risk.

## Materials and methods

### Overview

A case-control study was conducted where eligible men aged 50–80 years were recruited from Kingston General Hospital in Kingston, ON, Canada from 1997 to 1999. Eligible participants were either scheduled for prostate core biopsy or were visiting the urology clinic for other reasons and had normal age-specific prostate specific antigen (PSA) levels (≤3.5 in 50–60 age group, ≤4.5 in 60–70 age group, and ≤6.5 in 70–80 age group) and a normal digital rectal examination (DRE). Of 1288 men seen in clinic, 746 did not meet these criteria. Of the 542 eligible subjects, 71 (13%) were excluded due to diagnosis of prostate intraepithelial neoplasia, use of a hormonal medication, or missing questionnaire, and 56 declined to participate (10%). Participants were 414 men (76%) enrolled prior to knowledge of diagnosis who completed a questionnaire regarding demographic, lifestyle, medical, diet and occupational histories, and donated 15 mL of blood. After biopsy and pathology exams for 214 eligible biopsy subjects, 80 were diagnosed as incident primary prostate cancer cases, and the other 134 were included as “biopsy controls.” In addition, 200 eligible cancer-free men of the same age distribution who were diagnosed with a non-cancer urologic condition were enrolled as “urology controls,” for a total of 334 controls. Among these groups, 43 cases (54%), 102 biopsy controls (76%), and 68 urology controls (34%) consented to *MTHFR* genotype analysis. This study was approved by the Human Ethics Research Board at Queen's University and Kingston General Hospital.

### Dietary nutrient assessment

The dietary section of the questionnaire was adapted from Byers and colleagues' food frequency questionnaire (Byers et al., 1985) and requested information about intake of 62 food items in the time period two years prior to interview. These food items accounted for the majority of vitamin, fiber, fat, protein, and caloric intakes, and the reproducibility and validity of a similar questionnaire has been verified (Willett et al., 1988). Participants were also asked about vitamin or mineral supplements containing folate taken for six months or more. Results concerning prostate cancer risk associated with dietary patterns and organochlorine exposures were published previously (Walker et al., 2005; Aronson et al., 2010).

Reported food intakes were converted to average consumption per day and dietary nutrient levels were determined based on the average portion size, as described in Canada's Food Guide. Folate was the primary nutrient of interest, while vitamins B_2_ (riboflavin), B_6_, and B_12_, fat, energy, and methionine levels were calculated due to their possible roles as confounding factors. Specifically, food nutrient levels were determined using the 1991 Canadian Nutrient file (Nutrition Research Division, and Health Canada, 2001). Folic acid obtained from fortified grain products was not included since mandatory fortification did not start in Canada until 1998 and our data refer to prior to that year. Calculations of methionine content were based on the 2007 Canadian Nutrient file (Nutrition Research Division, and Health Canada, 2007) since the 1991 version did not contain these measurements. Data from the 2007 and 1991 Canadian Nutrient files were in agreement (excluding fortified grains) for most categories.

For categories containing more than one food item (e.g. “orange or grapefruit juice”), the Canada Food Statistics 1996 (Agriculture and Agri-Food Canada, and Statistics Canada, 2010) and the Canadian Food Value Chain Bureau Retail Sales data in 1996 (Agriculture and Agri-Food Canada, Food Value Chain Bureau, 2004) were used to estimate the average consumption of each food item within a food category (e.g., orange juice was weighted as 93% and grapefruit juice as 7% of the total nutrient value of “orange and grapefruit juice,” based on the average purchases of each item). The unstable nature of folate when cooked or heated was accounted for in the analysis since food preparation techniques impact nutrient levels. All components used to determine the nutrient values for the 62 food items are available upon request. Total nutrient levels were calculated for each individual based on their average consumption, and nutrient levels were adjusted to account for total caloric intake (Willett, 1998).

### Alcohol consumption assessment

Alcohol consumption was defined as one alcoholic drink = 12 oz of beer, 4 oz of wine, or 1.5 oz of liquor. Alcohol exposure variables were: age started drinking, years drinking, average total alcohol per week two years prior to study entry, average weekly consumption of beer, wine, and liquor separately two years prior to study entry, and lifetime alcohol intake.

### *MTHFR* C677T polymorphism analysis

Blood samples were stored at –80°C and were defrosted to room temperature for analysis. DNA isolation was performed using the QIAamp DNA Blood Mini Kit spin protocol (Qiagen, Valencia, CA), and PCR-RFLP analysis was based on methods described previously (Frosst et al., 1995; El-Sammak et al., 2004). Primers corresponding to the C677T site of the *MTHFR* gene had the exonic sequence of 5′-TGAAGGAGAAGGTGTCTGCGGGA-3′ and the intronic sequence of 5′-AGGACGGTGCGGTGAGAGTG-3′. The *MTHFR* C667T polymorphism was identified using the HinfI enzyme (Promega), which cleaves the DNA when the variant allele is present. The wildtype CC fragment showed a 198 base pair (bp) fragment when analysed by gel electrophoresis. The cleaved homozygous TT variant showed two fragments of 175 and 23 bp. The cleaved heterozygous CT variant produced three fragments of 198, 175, and 23 bp. To ensure quality control, PCR-RFLP results for a subset of gene fragments were verified against exact nucleotide sequences for these fragments, as sequenced by Cortec DNA Service Laboratories (Kingston, ON).

### Statistical analysis

Independent associations between folate intake, alcohol consumption, and *MTHFR* C677T genotype and risk of prostate cancer were estimated using unconditional logistic regression, with age included in all models. Folate intake was adjusted for energy intake, and was analysed continuously (per 100 μg/day), as tertiles based on intake among controls, and as ever use of a vitamin or mineral supplement containing folate for ≥6 months (yes/no). Intakes of vitamins B_2_, B_6_, and B_12_, and methionine were each analysed in these ways. Folate intake was also examined by Gleason score among cases, stratified into highly (score ≥7) and less aggressive (score 5–6) groups. Alcohol consumption was analysed as age started drinking, total alcohol intake per week (>5 drinks vs. ≤5), total beer, wine, and liquor per week (1–7 drinks and ≥8 drinks vs. <1 drink), and total lifetime alcohol intake (continuous per 1000 drinks, and as tertiles based on distribution among controls). Analyses of alcohol variables were performed excluding never drinkers (*n* = 68 cases and 292 controls included). *MTHFR* genotype was analysed with risk of the CT (heterozygous mutant) and TT genotypes (homozygous mutant) compared with the CC genotype (wildtype), both individually and combined as CT + TT in a dominant genetic model (Lewis, 2002). Potential confounders in addition to age were selected using a backward change-in-estimate (≥10%) method (Rothman and Greenland, 1998).

The joint effects of folate intake, alcohol consumption, and *MTHFR* genotype were estimated using unconditional logistic regression models adjusted for the same covariates as the main effect models, with combinations of low folate intake, low alcohol consumption, and the CC *MTHFR* genotype as the common reference groups. Low folate consumption was defined as ≤214.03 μg per day (with >214.03 defined as high), and low alcohol consumption was defined as ≤5 drinks per week (with >5 defined as high). Statistical significance of multiplicative and additive interactions was assessed from the likelihood ratio tests performed using the GENMOD procedure in SAS, which allows testing of these two types of interactions separately in general linear models (Littell et al., 2002). Analyses of interactions involving *MTHFR* genotype were performed using a dominant genetic model where the CT and TT genotypes were combined and compared to the CC genotype, as too few cases and controls displayed the TT genotype to use an additive gene model (Lewis, 2002).

Sensitivity analyses compared cases to a sub-group of controls excluding biopsy controls, and separately to controls excluding those with benign prostatic hyperplasia. Statistical analyses were performed using SAS (Version 9.2, SAS Institute, Cary, NC).

## Results

While most characteristics of cases and controls were similar (Table 1), controls had more cumulative smoking exposure and cases were more likely than controls to report strenuous physical activity as a teen. In terms of the exposures of interest, cases started drinking alcohol at a younger mean age (18 years old) than controls (almost 20 years old), and consumed more total alcohol per week on average than controls (Table 2). Consumption of folate was similar between cases and controls (Table 2). Consumption of vitamins B_2_, B_6_, B_12_, and methionine was similar (not shown).

**Table 1:** **Characteristics of study participants, *n* = 414 (80 cases; 334 controls)**.

**Characteristics**	**Cases *n* (%)**	**Controls *n* (%)**	***p-value***
Age (years), mean ± SD	65.1 ± 6.0	63.6 ± 6.9	0.08
**GLEASON SCORE**			
5	3 (4)	−	−
6	30 (38)	−	−
7	25 (31)	−	−
8	11 (14)	−	−
9	8 (10)	−	−
Missing	3 (4)	−	−
**MARITAL STATUS**			
Married	73 (91)	286 (86)	0.18
Other	7 (9)	48 (14)	−
**ANCESTRY**			
British	59 (74)	230 (70)	0.48
Other	21 (26)	100 (30)	−
**EDUCATION**			
Secondary or less	39 (49)	160 (48)	0.91
Post- secondary	41 (51)	173 (52)	−
**PERSONAL INCOME**			
Low	43 (54)	190 (57)	0.61
High	37 (46)	144 (43)	−
**BMI AT AGE 40 YEARS (kg/m^2^)**			
Mean ± SD	24.5 ± 0.3	25.0 ± 3.1	0.28
<23.68	29 (36)	113 (33)	0.64^*^
23.69–25.88	29 (36)	111 (33)	−
>25.88	22 (28)	110 (33)	−
**CURRENT SMOKING STATUS**			
No	65 (81)	293 (88)	0.13
Yes	15 (19)	41 (12)	−
Lifetime smoking pack-years among ever smokers, mean ± SD	30.6 ± 1.7	43.0 ± 6.1	0.06
**FIRST-DEGREE FAMILY HISTORY OF PROSTATE CANCER**			
No	61 (86)	270 (85)	0.87
Yes	10 (14)	47 (15)	−
**FIRST-DEGREE FAMILY BREAST CANCER HISTORY**			
No	70 (91)	279 (87)	0.40
Yes	7 (9)	40 (13)	−
**PHYSICAL ACTIVITY AS A TEEN**			
Little	20 (25)	133 (40)	0.012
Moderate	39 (49)	150 (45)	−
Strenuous	21 (26)	50 (15)	−
**VASECTOMY**			
No	58 (72.5)	240 (72)	0.90
Yes	22 (27.5)	94 (28)	−

* p for trend.

p-values < 0.05 were considered statistically significant.

**Table 2:** **Associations between folate intake, alcohol consumption, the *MTHFR* C677T polymorphism, and prostate cancer risk, *n* = 414 (80 cases; 334 controls)**.

	**Cases *n* (%)**	**Controls *n* (%)**	**OR (95% CI)**
FOLATE INTAKE^*^			
Folate(μg/day)			
Continuous (per 100 μg/day mean ± SD)	217.9 ± 5.7	220.6 ± 2.9	0.78 (0.47–1.30)
Tertiles			
≤197.54	26 (33)	111 (33.3)	1.00 (ref)
>197.54<234.99	25 (31)	112 (33.3)	0.85 (0.45–1.62)
≥234.99	29 (36)	111 (33.3)	1.03 (0.55–1.95)
Supplement containing folate			
<6 months	50 (62.5)	217 (65)	1.00 (ref)
≥6 months	30 (37.5)	117 (35)	1.15 (0.67–1.97)
**ALCOHOL CONSUMPTION^§^**			
Age starting drinking (per 1 year; mean ± SD)	18.5 ± 4.6	19.9 ± 6.9	0.95 (0.90–1.00)
Total years drinking (per 10 years; mean ± SD)	41.9 ± 12.5	41.2 ± 12.5	0.96 (0.83–1.10)
Total alcohol intake per week			
*Adjusted for age*			
≤5 drinks	22 (32)	133 (46)	1.00 (ref)
>5 drinks	46 (68)	159 (54)	1.75 (1.00–3.06)
*Adjusted for age, folate, MTHFR C677T genotype, and multiplicative folate-genotype interaction*			
≤5 drinks	8 (22)	68 (47)	1.00 (ref)
>5 drinks	29 (78)	78 (53)	3.22 (1.36–7.59)
Beer intake per week			
<1 drink	30 (44)	147 (50)	1.00 (ref)
1–7 drinks	27 (40)	111 (38)	1.23 (0.69–2.21)
≥8 drinks	11 (16)	34 (12)	1.69 (0.76–3.72)
Wine intake per week			
<1 drink	35 (51)	157 (54)	1.00 (ref)
1–7 drinks	24 (35)	112 (38)	0.95 (0.54–1.69)
≥8 drinks	9 (13)	23 (8)	1.71 (0.73–4.04)
Liquor intake per week			
<1 drinks	35 (52)	173 (59)	1.00 (ref)
1–7 drinks	25 (37)	103 (35)	1.21 (0.69–2.14)
≥8 drinks	8 (12)	16 (6)	2.27 (0.89–5.80)
Total lifetime alcohol intake			
Continuous (per 1000 drinks)	296.6 ± 166.9	176.1 ± 178.9	1.01 (1.00–1.03)
Tertiles			
Low	10 (19)	71 (33)	1.00 (ref)
Middle	16 (30)	72 (33)	1.31 (0.66–2.60)
High	27 (51)	73 (34)	2.08 (1.12–3.86)
***MTHFR* C677T POLYMORPHISM^§^**			
CC	22 (51)	72 (42)	1.00 (ref)
CT	19 (44)	86 (51)	0.72 (0.36–1.44)
TT	2 (5)	12 (7)	0.55 (0.11–2.65)
CT + TT vs. CC	21 (49)	98 (58)	0.70 (0.36–1.37)

* ORs adjusted for age, personal income, marital status, physical activity as a teen, family history of breast cancer.

§ ORs adjusted for age.

p-values < 0.05 were considered statistically significant.

No association with prostate cancer risk was apparent for dietary folate analysed continuously, by tertile, or according supplement use as the source, nor for vitamins B_2_, B_6_, B_12_, or methionine (folate only shown in Table 2). When cases were stratified by Gleason score, 57% had a score ≥7, and 43% had a score 5–6. Of those with Gleason score ≥7, 32% were in the lowest tertile of folate intake, while 12% with a score of 5–6 were in the lowest tertile, indicating lower folate with more prostate cancer progression (*p-trend* = 0.06).

In terms of alcohol consumption, also seen in Table 2, the highest tertile of lifetime total alcohol intake was associated with a doubling in prostate cancer risk (OR = 2.08; 95% CI: 1.12–3.86). Average weekly total alcohol consumption of greater than five drinks per week was associated with increased prostate cancer risk (OR = 1.75; 95% CI: 1.00–3.06). When further adjusted for folate intake, *MTHFR* C677T genotype, and the multiplicative interaction between the two, the magnitude of risk was even higher (OR = 3.22; 95% CI: 1.36–7.59).

In terms of *MTHFR* C677T genotype among consenting cases, 44% displayed the CT genotype, 5% displayed the TT genotype, and 51% displayed with wildtype CC genotype. Among genotyped controls, these numbers were 51, 7, and 42%, respectively. The CT and TT *MTHFR* genotypes were not associated with prostate cancer risk (Table 2).

Folate intake and alcohol consumption considered together are shown in Table 3. Combined high alcohol and low folate intakes were associated with increased prostate cancer risk (OR = 2.38; 95% CI: 1.01–5.57), although the interaction was not significant under multiplicative or additive models (Table 3). No multiplicative or additive effects of *MTHFR* genotype and folate intake were observed (Table 4). When the *MTHFR* genotype and alcohol consumption were investigated together, the OR for >5 compared to ≤5 drinks per week was 4.43 (95% CI: 1.15–17.05) within the CC genotype (Table 5). Sensitivity analyses comparing cases to controls excluding biopsy controls, and separately to controls not diagnosed with benign prostatic hyperplasia, found results similar to the overall analysis.

**Table 3:** **Folate intake, alcohol consumption, and prostate cancer risk (68 cases; 292 controls)**.

**Folate intake**	**Alcohol consumption**	**Cases *n* (%)**	**Controls *n* (%)**	OR (95% CI)^*^
Low	Low	10 (15)	68 (23)	1.00 (ref)
Low	High	26 (38)	76 (26)	2.38 (1.01–5.57)
High	Low	12 (18)	65 (22)	1.16 (0.44–3.09)
High	High	20 (29)	83 (28)	1.67 (0.70–4.01)
*p-interaction (multiplicative)*				0.80
*p-interaction (additive)*				0.69

* ORs adjusted for age, personal income, marital status, physical activity as a teen, and family history of breast cancer.

p-values < 0.05 were considered statistically significant.

**Table 4:** **Folate intake, *MTHFR* C677T genotype, and prostate cancer risk (43 cases, 170 controls)**.

**Folate intake**	***MTHFR* genotype**	**Cases *n* (%)**	**Controls * n* (%)**	OR (95% CI)^*^
Low	CC	11 (26)	33 (19)	1.00 (ref)
Low	CT + TT	11 (26)	47 (28)	0.85 (0.31–2.37)
High	CC	11 (26)	39 (23)	1.06 (0.37–3.03)
High	CT + TT	10 (23)	51 (30)	0.73 (0.26–2.08)
*p-interaction (multiplicative)*				0.73
*p-interaction (additive)*				0.76

* ORs adjusted for age, personal income, marital status, physical activity as a teen and family history of breast cancer.

**Table 5:** **Alcohol consumption, *MTHFR* C677T genotype, and prostate cancer risk (37 cases; 146 controls)**.

**Alcohol consumption**	***MTHFR* genotype**	**Cases *n* (%)**	**Controls *n* (%)**	**Age-adjusted OR (95% CI)**
Low	CC	3 (8)	29 (20)	1.00 (ref)
Low	CT + TT	5 (14)	39 (27)	1.25 (0.28–5.65)
High	CC	14 (38)	31 (21)	4.43 (1.15–17.05)
High	CT + TT	15 (41)	47 (32)	3.10 (0.83–11.65)
*p-interaction (multiplicative)*				0.51
*p-interaction (additive)*				0.58

p-values < 0.05 were considered statistically significant.

## Discussion

In this study of incident primary prostate cancer cases and clinic-based controls, all of whom were men with normal PSA and DRE results, independent effects of folate intake and *MTHFR* genotype on prostate cancer risk were not observed. We did observe that total lifetime alcohol consumption increased prostate cancer risk two-fold among men in the highest consumption tertile. Further, consumption of more than five alcoholic drinks per week increased prostate risk over two-fold among men with low folate intake and over four-fold among men with the CC genotype. Average weekly alcohol consumption was also associated with increased prostate cancer risk when folate intake, *MTHFR* C677T genotype, and their multiplicative interaction were accounted for. We observed no gene-environment interaction between the *MTHFR* C677T polymorphism and folate intake.

Our findings for the effects of folate intake on prostate cancer risk are consistent with five case-control studies (Vlajinac et al., 1997; Weinstein et al., 2003; Johansson et al., 2008; Zhang et al., 2009; Collin et al., 2010b) and three cohort studies (Stevens et al., 2006; Weinstein et al., 2006; Beilby et al., 2010). Our results are inconsistent with two case-control studies reporting decreased risk with higher dietary folate intake (Pelucchi et al., 2005; Shannon et al., 2009), and a prospective study and randomized clinical trial both finding increased risk with higher intakes (Hultdin et al., 2005; Figueiredo et al., 2009). Inconsistent findings from existing studies may be in part due to failure to take interactions and stage and/or grade of prostate cancer at diagnosis into account. Increasing evidence supports a dual role for folate intake in prostate carcinogenesis wherein both high and low intakes may have deleterious effects at different stages in tumor progression (Stevens et al., 2006; Shannon et al., 2009; Collin et al., 2010a,b; Bistulfi et al., 2011).

The distribution of *MTHFR* genotypes among both cases and controls in our study was similar to the estimated population distribution of approximately 40, 50, and 10% prevalence for the CC, CT, and TT genotypes, respectively (Lathrop Stern et al., 2000). Our results for the CT and TT *MTHFR* genotypes were null, although the ORs below one that we observed are consistent with one meta-analysis (Bai et al., 2009), while another shows no association between the *MTHFR* C677T polymorphism and prostate cancer risk (Collin et al., 2009).

Although this is a relatively small study that lacked power to detect most interactions, our finding of an over two-fold increase in prostate cancer risk for the interaction of low folate and high alcohol intake is consistent biologically with prostate carcinogenesis resulting from folate deficiency (Bistulfi et al., 2010). Other epidemiologic evidence for a folate-alcohol interaction is conflicting (Weinstein et al., 2003; Pelucchi et al., 2005; Stevens et al., 2006; Shannon et al., 2009). One study has examined a *MTHFR*-folate genotype interaction in prostate cancer, finding that the C677T polymorphism increases risk at high plasma folate levels (Van Guelpen et al., 2006). Our finding of an over four-fold increase in prostate cancer risk associated with alcohol consumption among men with the CC *MTHFR* genotype indicates a potentially strong gene-environment interaction between these two factors that has not been investigated in any other study we are aware of.

Strengths of this study include rigorous exclusion criteria and confirmation of all controls as having normal age-specific PSA levels. This decreased selection bias and ensured that the control group was not contaminated with latent prostate cancer. All men visited the same urologists in a single hospital and resided in the same geographic catchment area. Because all participants were recruited and completed the questionnaire prior to knowledge of disease status, differential recall error, and interviewer bias were avoided in this study. The food frequency questionnaire was highly detailed, reproducible, and valid, and was designed to represent average diet two years prior to study enrollment. The comprehensive statistical analysis of folate, B vitamins, and methionine, using energy-adjustment and three types of input variables also reduced the possibility of exposure misclassification. Questioning about average alcohol intake two years prior (relatively recent) reduced the possibility of recall error (Middleton Fillmore et al., 2009), and the comprehensive indicators of alcohol consumption reduced the possibility of exposure misclassification.

Dietary folate consumption among our study population was measured prior to 1998, when folate fortification of white wheat flour became mandatory in Canada and the United States. Dietary folate consumption and folate status of our study population are likely lower than that of the more recent general Canadian and US populations (Baily et al., 2010; Colapinto et al., 2011). Our results are relevant since assessing lower folate consumption in these countries may be more difficult now because of fortification. However, low variation in our observed range of folate intakes may have limited our ability to detect both main effects and interactions with folate, since folate intake in the first and third tertiles differed by only 38 μg in our study.

In summary, we found that alcohol consumption is associated with increased prostate cancer risk, with higher risk among men with low folate intake and among men with the CC *MTHFR* genotype. Taking into account potential combined effects and interactions is important for future studies, and may in part explain inconsistent findings to date. Future work should also capture a greater range of folate intakes to determine the true role of folate in prostate carcinogenesis, and whether this is role is dualistic in nature, to inform public policy regarding folate fortification.

### Conflict of interest statement

The authors declare that this research was conducted in the absence of any commercial or financial relationships that could be construed as a potential conflict of interest.
